# Three viruses of the bovine respiratory disease complex apply different strategies to initiate infection

**DOI:** 10.1186/1297-9716-45-20

**Published:** 2014-02-18

**Authors:** Jana Kirchhoff, Sabine Uhlenbruck, Katherina Goris, Günther M Keil, Georg Herrler

**Affiliations:** 1 Institute of Virology, University of Veterinary Medicine Hannover, Hannover, Germany; 2 Institute of Molecular Biology, Friedrich-Loeffler-Institut, Greifswald - Insel Riems, Germany

## Abstract

Bovine respiratory disease complex (BRDC) is the major cause of serious respiratory tract infections in calves. The disease is multifactorial, with either stress or reduced immunity allowing several pathogens to emerge. We investigated the susceptibility of bovine airway epithelial cells (BAEC) to infection by the three major viruses associated with the BRDC: bovine respiratory syncytial virus (BRSV), bovine herpesvirus type 1 (BHV-1) and bovine parainfluenza virus type 3 (BPIV3). For this purpose, two culture systems for well-differentiated BAEC were used: the air-liquid interface (ALI) system, where filter-grown BAEC differentiate into a pseudostratified respiratory epithelium and precision-cut lung slices (PCLS) where BAEC are maintained in the original tissue organisation. Comparative infection studies demonstrated that entry and release of BPIV3 occurred specifically via the apical membrane with ciliated cells being the major target cells. By contrast, airway epithelial cells were largely resistant to infection by BHV-1. When the epithelial barrier was abolished by opening tight junctions or by injuring the cell monolayer, BHV-1 infected mainly basal cells. Respiratory epithelial cells were also refractory to infection by BRSV. However, this virus infected neither differentiated epithelial cells nor basal cells when the integrity of the epithelial barrier was destroyed. In contrast to cells of the airway epithelium, subepithelial cells were susceptible to infection by BRSV. Altogether, these results indicate that the three viruses of the same disease complex follow different strategies to interact with the airway epithelium. Possible entry mechanisms are discussed.

## Introduction

Virus infections of the respiratory tract are the most common cause of viral diseases worldwide ranging from common colds to life-threatening pneumonia [1]. A large number of both RNA and DNA viruses uses the respiratory tract to initiate host infection. Infection may be restricted to or most evident in certain sections of the airway system such as trachea, bronchi or alveoli. For some viruses, the respiratory tract may just serve as a primary entry site from where infection spreads to other organs or tissues. All these viruses encounter the respiratory epithelium as a primary barrier against invading pathogens. This barrier is composed of differentiated epithelial cells that have different functions. An important defence strategy is the mucociliary clearance system. While some epithelial cells are specialized to produce and release mucins, other cells are equipped with cilia that enable them to contribute to the transport of the mucus out of the respiratory tract.

Viruses differ in their ability to infect the differentiated respiratory epithelial cells. Productive infection by human influenza viruses has been reported to preferentially occur in mucus-producing cells [2]. Other viruses, e.g. parainfluenza virus 3 (PIV3), may have a preference for ciliated cells [3]. Characteristic features of differentiated epithelial cells such as ciliary activity are not maintained in any of the available immortalized cell lines. Analysis of such cells requires primary cell cultures. When tracheal or bronchial epithelial cells are grown on filter supports under air-liquid interface (ALI) conditions, they differentiate and acquire properties of specialized cells, i.e. mucus production or ciliary activity [4,5]. Another culture system for differentiated respiratory epithelial cells are precision-cut lung slices (PCLS), where the epithelial cells are maintained in their original setting. In addition to mucus production and ciliary activity, this culture system provides another characteristic feature of the airway, bronchoconstriction. As submucosal cells are also present in PCLS, they can be included in the investigation [6-8].

Bovine respiratory disease complex (BRDC) is a leading cause of morbidity and mortality in feedlot cattle. The disease is considered as a multifactorial disorder caused by a combination of viral and bacterial pathogens together with environmental risk factors. The most important viral pathogens associated with BRD are bovine respiratory syncytial virus (BRSV), bovine parainfluenza virus 3 (BPIV3) and bovine herpesvirus 1 (BHV-1), a member of the subfamily *Alphaherpesvirinae*[9-12]. BRSV and BPIV3 are paramyxoviruses that use the fusion protein F on the cell surface to mediate fusion of the viral envelope with the cellular membrane and thus to get the viral genome into the cell. Prior to fusion, the virions attach to the cell surface. Attachment of PIV3 is accomplished by another surface protein, the HN protein which interacts with sialic acid-containing cellular glycoconjugates and thus binds to cells in a sialic acid-dependent manner [13,14]. Attachment of human RSV (HRSV) can be mediated by the surface protein G that binds to glycosaminoglycan structures [15] as well as by the fusion protein F that may bind to both glycosaminoglycans as well as to a specific protein receptor [16,17]. Nucleolin has recently been reported to promote infection though the authors did not exclude the possibility that another so far unknown protein may serve as a receptor for entry of HRSV [18]. No data on receptors for BRSV have been reported yet. Alphaherpesviruses like BHV-1, herpes simplex virus 1 (HSV-1) or pseudorabies virus use the surface glycoprotein gC for initial attachment to heparan sulphate structures. For entry, virions have to bind to a specific receptor (nectin-1 or herpesvirus entry mediator (HVEM)) which is accomplished by the surface protein gD. The subsequent fusion reaction is mediated by the combined action of the surface proteins gB/gD/gH/gL [19,20].

We have reported recently that well-differentiated respiratory epithelial cells are susceptible to infection by BPIV3 whereas they are rather resistant to infection by BRSV [7]. Here we have included another important virus of the BRDC, BHV-1 and analysed virus entry into and release from differentiated respiratory epithelial cells in more detail. Our results show that the three viruses have developed different strategies to infect the cells of the airway epithelium.

## Materials and methods

### Viruses

The generation of recombinant BRSV expressing green fluorescent protein (BRSV-GFP) and preparation of viral stocks has been described in detail elsewhere (7; BRSV ATue51908 was kindly provided by Karl-Klaus Conzelmann, Max-von-Pettenkofer-Institut, Munich). BPIV3 was provided by Friedrich-Loeffler-Institut (Insel Riems, Germany). BPIV3 stocks were prepared in KOP-R cells [7]. BHV-1-GFP was generated from the strain BHV-1/Aus12 as described previously [21] and propagated in KOP-R cells. For this purpose, cells were grown in 75 cm^2^-flasks till 80% confluence and then infected with an MOI of 0.1 for 2 h. After 1–3 days a cytopathic effect was observed and supernatants were collected and centrifuged by low speed centrifugation (2000 × *g*). All virus stocks were frozen in liquid nitrogen and stored at -80 °C.

### Cell cultures

MDBK (Madin-Darby bovine kidney cells; kindly provided by Wolfgang Garten, Philipps-Universität Marburg, Marburg, Germany), Vero cells (African Green Monkey cells; ATCC, CCL-81) as well as KOP-R cells (bovine oropharynx tissue, RIE 244; Friedrich-Loeffler-Institut, Insel Riems, Germany) were cultured in Dulbecco’s modified Eagle medium (DMEM) supplemented with 5 or 10% fetal calf serum (Biochrom AG, Berlin). All cell lines were maintained in 75-cm^2^ culture flasks at 37 °C and 5% CO_2_ and passaged twice a week.

### ALI cultures

Primary BAEC (bovine airway epithelial cells) were derived from bovine bronchi of 6–8 months old calves and epithelial cells were prepared as described previously [5,7]. Briefly, cells were seeded on collagen coated, semipermeable membrane supports (Greiner, 24 well, 0.4-μm pore size) at a density of 2.5 × 10^5^ cells per filter support. After BAEC had reached confluence, the apical medium was removed and cells were cultured under ALI conditions for at least 3 weeks to establish a polarized and differentiated epithelium. Prior to infection, the transepithelial electrical resistance (TEER) was determined to verify an intactness of the epithelial barrier. For this purpose, a Millicell ERS-2 Volt-Ohm meter (Millipore Corporation, Billerica, MA, USA) was used. To obtain the actual resistance of the epithelial cell layer, the resistance value of a cell-free filter support was subtracted from the TEER values determined across the filter-grown cultures. TEER values were above 400 Ω ∙ cm^2^ prior to infection.

### PCLS

PCLS were prepared from the *lobus accessorius* of 6–8 months old calves as described previously [6,7]. Lobes were filled with low-melting agarose (agarose LM GQT, GERBU, Gaiberg, Germany). After the agarose had solidified, cylindrical portions containing a section of an airway were stamped out. Using a Krumdieck tissue slicer (TSE systems, model MD4000-01) slices, 250 μm thick, were generated and incubated in 1 mL of RPMI 1640 medium (Invitrogen/Gibco, Germany) in 24-well plates at 37 °C and 5% CO_2_. The viability of the slices was determined by screening for ciliary activity using a light microscope (Zeiss Axiovert 35).

### Viral inoculation

BAEC were washed extensively with phosphate-buffered saline (PBS) to remove secreted mucus. Subsequently, the virus suspension, diluted in DMEM, was added at 100 μL per filter to the cells for 2 h (BPIV3, BHV-1-GFP) or 3 h (BRSV-GFP) at 37 °C and 5% CO_2_. For infection of the cells from the basolateral surface, filters were inverted for the duration of infection. After unbound virus had been removed by washing, the cells were further incubated at 37 °C for 1 to 3 days.

To open tight junctions, the cells were washed thrice with Ca^2+^/Mg^2+^-free PBS and then apically treated with Ca^2+^/Mg^2+^-free PBS containing 0.1 M EGTA (AppliChem, Darmstadt) at 37 °C. After 10 min, EGTA was removed and cells were infected with either BHV-1-GFP or BRSV-GFP. TEER was determined before and after EGTA treatment.

PCLS were washed three times with PBS to remove mucus and then infected with virus (10^5^ FFU/mL) diluted in 500 μL of RPMI medium. After 2 h (BHV-1-GFP, BPIV3) or 3 h (BRSV-GFP), the inoculum was removed and the slices were washed twice with PBS followed by the addition of 1 mL of RPMI medium. Slices were incubated for 1–7 days in 5% CO_2_ at 37 °C. To analyze the importance of sialic acids for BPIV3 infection, the slices were washed with PBS followed by incubation with 100 mU/PCLS of neuraminidase (NA) type V from *Clostridium perfringens* (Sigma/Aldrich, Munich) for 1 h at 37 °C. After removal of NA, the slices were rinsed in PBS prior to virus infection.

To assess viral shedding, supernatants of apically infected ALI cultures were taken post-infection over a period of 1 week. For this purpose, 100 μL aliquots of pre-warmed EMEM were applied to the apical surface of the filter inserts and harvested after a 30 min incubation period at 37 °C. For basolateral virus release, 500 μL of the basolateral medium was collected and replaced by fresh medium. Supernatants were combined with 10 × viral stabilizing solution (1 M MgSO_4_, 0.5 M HEPES, pH 7.5), quick-frozen on dry ice and stored at -80 °C. TEER was determined during the course of the experiment. In the case of PCLS, 100 μL of the supernatant was collected at each time point and replaced by the same amount of RPMI to avoid differences in virus concentration. For each virus, cultures from at least 3 animals were used and experiments were performed in duplicate.

### Fluorescence microscopy

Cells grown on membrane filters and PCLS were fixed for 20 min at room-temperature with 3% paraformaldehyde in PBS. Fixed cells were washed twice with 0.1 M glycine in PBS and then permeabilized with 0.2% Triton X-100 diluted in PBS for 10 min. All antibodies were diluted in PBS containing 1% bovine serum albumin at room temperature. BPIV3-infected cells were stained with a polyclonal antiviral antiserum (VMRD, caprine origin) followed by a FITC-labeled secondary antibody (Sigma-Aldrich). Cilia were stained by using a Cy3-labeled monoclonal antibody against β-tubulin (Sigma-Aldrich). Basal cells were visualized by a monoclonal mouse anti-human CD142 antibody (Serotec) and a primary antibody specific for TTF-1 (clone BGX-397A mouse monoclonal, BioGenex) was used to stain pneumocytes type II. Nuclei were visualized with DAPI (4′, 6′-diamidino-2-phenylindole) which was added to the apical surface of the cells and removed after incubation for 15 min (37 °C) followed by three washing steps with PBS. After washing, cells were embedded in Mowiol resin and photomicrographs were generated using a Leica TCS SP5 AOBS confocal laser scanning microscope. For image processing, Adobe Photoshop (Version 10.0, Adobe Systems, USA) was used.

### Immunoplaque assay

One day prior to infection, MDBK (BPIV3, BHV-1-GFP) or Vero cells (BRSV-GFP) were grown to confluence in 96 well plates and then exposed to serial tenfold dilutions of virus suspensions in duplicate. For virus adsorption, cells were incubated at 37 °C for 2 h (BPIV3, BHV-1-GFP) or 3 h (BRSV-GFP). After inoculation, cells were overlaid with 3% methylcellulose and plaques were counted 2 (BHV-1-GFP, BPIV3) or 3 (BRSV-GFP) days post-infection. In case of BPIV3, the cells were stained for virus antigen. The viral titer is indicated in foci-forming units per mL (FFU/mL).

## Results

### Virus infection of ALI cultures

**Figure 1:**
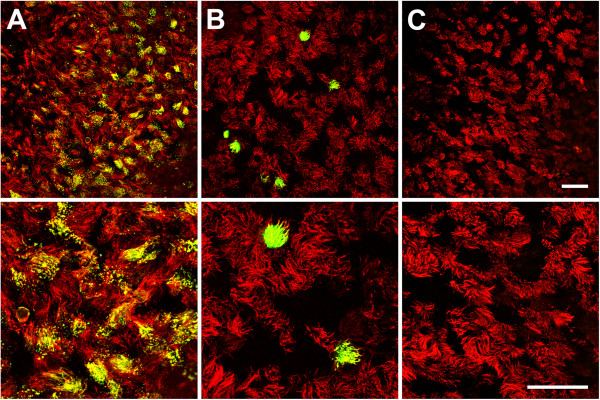
**Infection of well-differentiated BAEC by BPIV3 BRSV-GFP and BHV-1-GFP at different magnifications. A)** BPIV3 was applied to the apical surface of ALI cultures (MOI~0.1) for 2 h. At 2 dpi, cultures were fixed and virus-infected cells were detected by immunostaining (green). **B)** BRSV-GFP was inoculated for 3 h and the cultures were fixed 3 dpi. Infected cells were detected by GFP expression. **C)** BHV-1-GFP was applied at an MOI of 0.1 to the apical side of ALI cultures for 2 h. 2 days later, cultures were fixed. Cilia were visualized by staining against β-tubulin (red). Scale bars = 50 μm.

**Figure 2:**
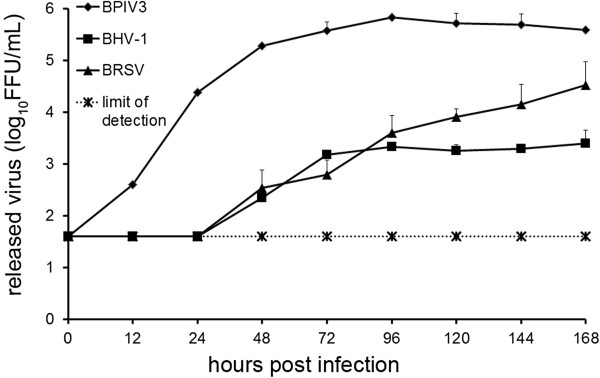
**Infection of ALI cultures by BPIV3, BRSV-GFP and BHV-1-GFP evaluated by virus titration.** ALI cultures were mock infected or infected by any of the three viruses. Up to 7 dpi, virus shed from the apical or basolateral surface was collected at different times pi. Titration was performed on Vero (BRSV-GFP) or MDBK (BPIV3, BHV-1-GFP) cells. Shedding was polarized and occurred from the apical side. Experiments were performed on lungs derived from 3 animals in duplicate. Indicated are mean values + standard deviation. Control values were not above detection level (40 FFU).

The three viruses associated with the bovine respiratory disease complex were compared for their efficiency to infect differentiated respiratory epithelial cells. For this purpose, BAEC were grown for about four weeks under ALI conditions to build up a polarized and pseudostratified epithelium. Cells were infected by BPIV3, BRSV-GFP or BHV-1-GFP at an MOI of 0.1 and analyzed two (BPIV3 and BHV-1-GFP) or three (BRSV-GFP) days post infection (dpi) for the presence of infected cells. As described in earlier studies [7], BPIV3 efficiently infected the respiratory epithelium. Co-staining of β-tubulin indicated that ciliated cells were the target cells (Figure 1A). On the other hand, infection by BRSV-GFP was very inefficient (Figure 1B); only a few infected cells were detectable and those cells were identified as ciliated cells using an antibody directed against β-tubulin. Only occasionally non-ciliated cells were infected by BRSV-GFP. When BHV-1-GFP was applied to the apical surface, the epithelium was found to be refractory to infection (Figure 1C). Not a single infected cell was detectable at two dpi; if the incubation time was increased up to five days, only occasionally infected cells were found. The same result was obtained when the titer was enhanced tenfold and infection was monitored up to five days. Only when an MOI of 10 was applied, a few cells were infected and appearance of infected foci suggested that infection spread to adjacent cells (see Additional file 1). Infection of BAEC by the three bovine viruses was also evaluated by determining the kinetics of virus production (Figure 2). Apical release of infectious BPIV3 was detected at 12 hours post infection (hpi) and increased exponentially by 24 hpi; the maximum infectivity titer of ~7 × 10^5^ FFU/mL was determined at 96 hpi. BRSV-GFP was detectable in the supernatant only at 48 hpi and the maximum titer of 3 × 10^4^ FFU/mL was reached after seven days. The apical release of infectious BHV-1-GFP was as slow as that of BRSV-GFP but proceeded only to a maximum titer of 2.5 × 10^3^ FFU/mL. Basolateral release of all three viruses was below detection level. For any of the three viruses, we did not observe a general decrease in the transepithelial resistance values during the course of the experiment. Despite some fluctuation of individual TEER values post-infection and variability between individual trans-wells, apical infection of differentiated cultures did not affect the integrity of the epithelium (see Additional file 2A).

**Figure 3:**
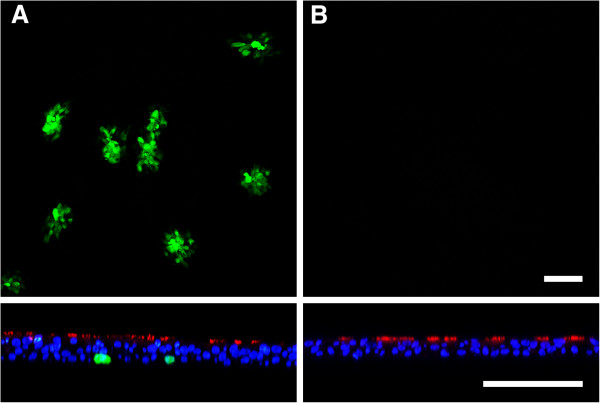
**BHV-1-GFP infection of the basolateral surface. A)** Cells were infected at the basolateral surface by exposing the inverted filter membrane to BHV-1-GFP at an MOI of 1 for 2 h. **B)** As a control, inverted filters were infected by BRSV-GFP for 3 h. Infection was monitored 1 dpi (BHV-1-GFP) or 3 dpi (BRSV-GFP) by staining for β-tubulin (red) and DAPI (blue). Virus-infected cells were detected by GFP expression. Lower panels show vertical sections. Scale bars = 100 μm.

Since BRSV-GFP and BHV-1-GFP were not able to establish an efficient infection of BAEC when applied from the apical side, we analyzed whether they could initiate infection from the basolateral side of the cells. For this purpose, the device containing the membrane was turned upside down. Under these conditions, BHV-1-GFP was able to infect BAEC as indicated by GFP expression after a 24 h incubation time. The efficiency of infection could be enhanced by increasing the MOI tenfold (Figure 3A). In contrast, BRSV-GFP was not able to establish an infection after basolateral inoculation (Figure 3B).

**Figure 4:**
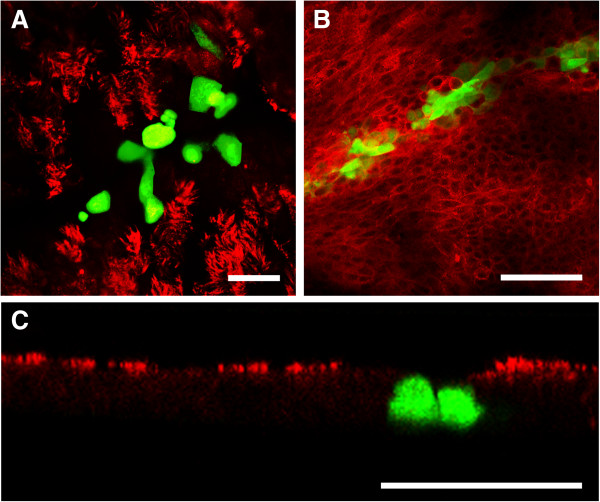
**Effect of mechanical damage of ALI cultures on BHV-1-GFP infection.** BHV-1-GFP was applied to the apical surface of BAEC after the pseudostratified epithelium had been injured with a sterile needle. At 1 dpi, cells were stained for β-tubulin **(A, C)** to visualize cilia or **(B)** CD142 to detect basal cells (both in red). **C)** shows a vertical section of injured epithelium. Scale bars = 25 μm in **(A)**, 100 μm in **(B)** and **(C)**.

The results presented above indicate that BHV-1 - but not BRSV-GFP is able to infect BAEC from the basal side. To rule out that the pore-size of the filter membrane and the size and morphology of the virions prevented access to the basal side, we used an epithelium injury model to investigate infection. Well-differentiated BAEC were mechanically injured with a sterile needle and subsequently infected by either BHV-1-GFP or BRSV-GFP from the apical surface for two or three h. As shown in Figure 4A, infection occurred exclusively within the region of injury and the underlying cells are the site of infection (Figure 4C). Further staining with anti-CD142 which is specifically expressed on bronchial epithelial basal cells [22] showed that BHV-1-GFP infected this cell type (Figure 4B). In contrast, in case of BRSV-GFP, hardly any cells at the injury site expressed GFP (data not shown). As shown above, a few infected cells were detected in the undamaged region; these cells were identified as ciliated cells by staining for β-tubulin.

**Figure 5:**
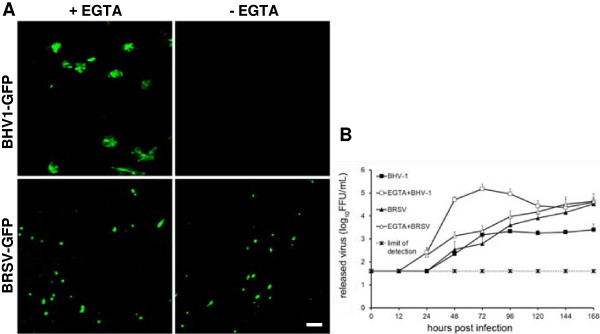
**Effect of EGTA treatment of BAEC on BHV-1-GFP infection.** Well-differentiated BAEC were treated with 0.1 M EGTA for 10 min or control buffer (PBS). Cells were then exposed to BHV-1-GFP or BRSV-GFP (MOI = 0.1) for 2 h. At 2 dpi (BHV-1-GFP) or 3 dpi (BRSV-GFP), the slices were fixed. Virus-infected cells were visualized by GFP expression **(A)**. Scale bar = 100 μm. Apical release of the viruses pretreated with (open symbols) or without (closed symbols) EGTA. Average and standard deviation from 3 animals (n = 2 each) are shown **(B)**.

To further analyze BHV-1 entry into BAEC, EGTA was used to transiently open the tight junctions. Cells were treated with 0.1 M EGTA for 10 min prior to infection with either BHV-1-GFP or BRSV-GFP. In contrast to untreated cultures, TEER values dropped to the baseline after EGTA treatment (see Additional file 2B). As shown in Figure 5A, pretreatment with EGTA enabled BHV-1-GFP entry from the apical side as indicated by GFP-positive foci distributed all over the filter-grown cells. By contrast, the disruption of the epithelial barrier had no effect on the efficiency of BRSV-GFP infection or on the cell type infected by this virus. Virus titration revealed that the yield of BHV-1-GFP released into the supernatant was increased up to 200 fold at 48 hpi. In case of BRSV-GFP, only low amounts of infectious virus were released into the supernatants and the final titer determined was similar (3.3 × 10^4^ vs. 4.3 × 10^4^ FFU/mL after one week) in the untreated and EGTA-treated samples. However, infection proceeded faster when the cells had been pretreated with EGTA (Figure 5B). The different efficiencies of BRSV-GFP and BHV-1-GFP in the infection of EGTA-treated ALI cultures were also evident in the recovery of the transepithelial resistance which took 48 h in the case of BRSV-GFP and 120 h in the case of BHV-1-GFP.

### Virus infection of precision-cut lung slices

**Figure 6:**
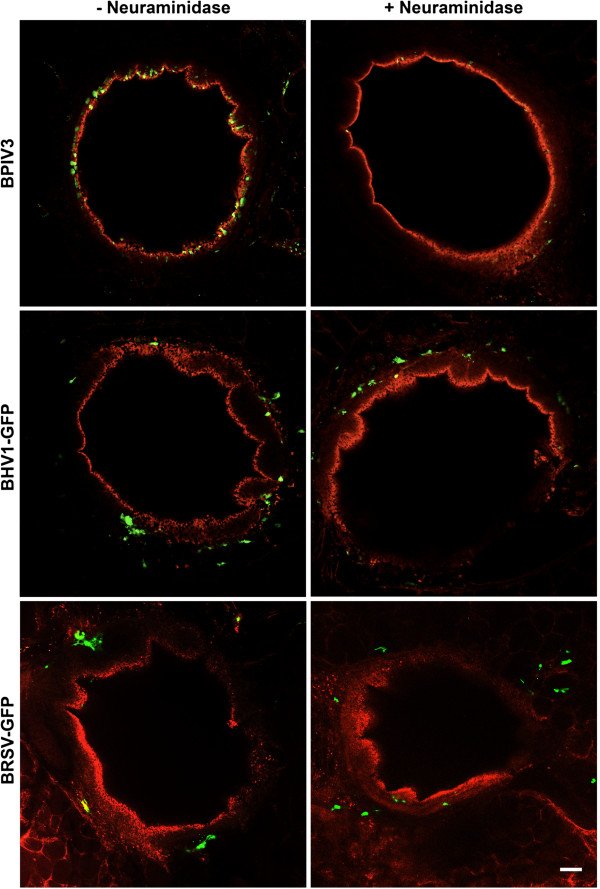
**BPIV3, BHV-1-GFP and BRSV-GFP infection of PCLS before or after neuraminidase treatment.** PCLS were first incubated for 1 h with or without 100 mU/PCLS neuraminidase type V and then infected with BPIV3 or BHV-1-GFP with 10^5^ FFU/mL for 2 h or with BRSV-GFP for 3 h. At 2 dpi, the slices were fixed and in case of BPIV3 stained for virus antigen (green). Cilia were visualized by staining against β-tubulin (red). Scale bar = 100 μm.

**Figure 7:**
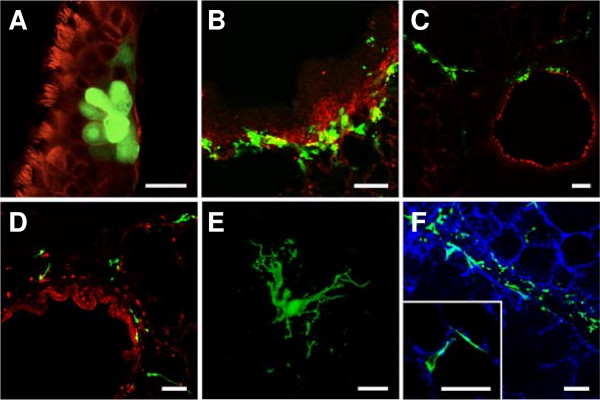
**BHV-1-GFP and BRSV-GFP infection of PCLS.** One day after preparation, PCLS were inoculated with BHV-1-GFP for 2 h or BRSV-GFP for 3 h (10^5^ FFU/mL). BHV-1-GFP infection **(A-C)** was stopped after 2 days **(A, B)** or after one week **(C)** and cells were stained for β-tubulin **(A, C)** to detect cilia or with anti-CD-142 for detection of basal cells (**B**, both in red). BRSV-GFP infection **(D-F)** was stopped after 3 days **(D, E)** or one week **(F)** and slices were stained for β-tubulin (red) and DAPI (blue). **(D)** BRSV-infected cells detected in the peribronchiolar connective tissue; **(E)** a characteristic shape of a BRSV-GFP-infected cell in the submucosal region. **F)** BRSV-GFP-infected cells within the peripheral part of the slice. The insert shows infected cells within the epithelium lining the alveolar lumen. GFP-expressing cells indicate virus-infected cells. Scale bars = 25 μm in **(A + E)** and 100 μm in **(B-D, F)**.

PCLS provide an alternative culture system for differentiated respiratory epithelial cells and have the advantage that they contain BAEC in the original setting. Moreover, connective tissues and alveolar regions are present within these slices as well as immunomodulatory cells. To analyze the infection strategies of BPIV3, BRSV-GFP as well as BHV-1-GFP, PCLS were prepared from the lungs of 6–8 months old calves. For infection studies, only those slices were used, that – upon microscopic inspection - did not show any loss of the ciliary activity. PCLS were infected with the same infectious dose (10^5^ FFU/mL) of any of the three viruses. The patterns of infected cells were quite different. BPIV3 exclusively infected ciliated cells of the respiratory epithelium, whereas BHV-1-GFP-infected cells were located underneath the uppermost epithelial cell layer (Figures 6 and 7A).

It is known that sialic acids serve as receptor determinants for entry of BPIV3. To determine the importance of sialic acids in the infection of differentiated respiratory epithelial cells by BPIV3, slices were treated with neuraminidase from *C. perfringens* prior to infection. As shown in Figure 6, desialylation almost completely abolished infection confirming that infection of differentiated respiratory epithelial cells – like that of immortalized cells (data not shown) - is sialic acid-dependent. Infection of the slices by BHV-1-GFP or BRSV-GFP was not affected by neuraminidase treatment. Staining for CD 142 revealed that basal cells, the progenitor cells of the differentiated respiratory epithelial cells, are primarily infected by BHV-1-GFP (Figure 7B). When the incubation time was increased to one week, infection spread to the periphery of the slice (Figure 7C). In contrast, basal cells were not targeted by BRSV-GFP. The latter virus predominantly infected subepithelial cells within the peribronchiolar connective tissue at three dpi and only a low number of ciliated cells were GFP-positive (Figure 7D). The morphologies of at least some infected cells in the submucosal area resembled those of dendritic cells (Figure 7E). After one week of incubation, BRSV-infected cells were also detected in the peripheral part of the slice comprising the alveoli (Figure 7F). Incubation with anti-TTF-1 (thyroid transcription factor -1), which stains nuclei of type II pneumocytes revealed that both, TTF-1 positive and negative cells, were infected. Stained cells were cuboidal in shape and found at the intersections of two or more alveoli whereas infected cells that were not stained by anti-TTF-1 had a flattened morphology (see Additional file 3).

**Figure 8:**
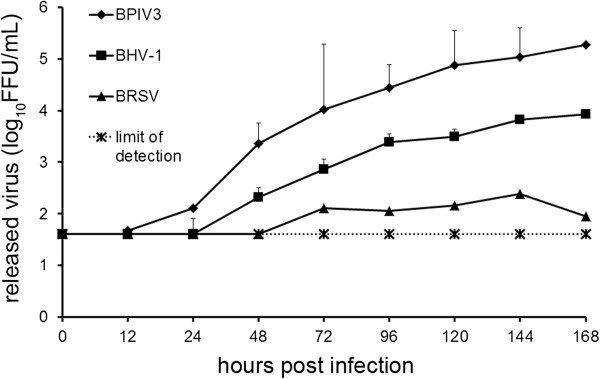
**Infection of PCLS by BPIV3, BRSV-GFP and BHV-1-GFP.** PCLS were mock-infected or infected by any of the three viruses (10^5^ FFU/mL). Up to 7 dpi, virus release into the supernatants was determined by titration on Vero (BRSV-GFP) or MDBK (BPIV3, BHV-1-GFP) cells. Experiments were performed with samples from 3 animals in duplicate. Indicated are mean values + standard deviation. The detection limit was set to 40 FFU.

The efficiency of infection was also determined with respect to infectious progeny virus released into the culture medium. For this purpose, supernatants were collected at different time points after infection. As shown in Figure 8, BPIV3 reached a viral titer of 2.3 × 10^3^ FFU/mL after 48 h, which increased to 2 × 10^5^ FFU/mL by day seven post infection (pi). Infection of PCLS by BRSV-GFP resulted in titers in the supernatant that were hardly above the detection level. Virus release of BHV-1-GFP was intermediate with titers that were 5 - to 20-fold lower than those determined for BPIV3. Consistent with the differences in the amount of virus released into the supernatant, there was also a difference in the kinetics of virus release: BPIV3 was detectable in the supernatant at 12 hpi followed by BHV-1-GFP at 24 hpi; BRSV-GFP was released into the supernatant not until 48 hpi.

## Discussion

Culture systems for differentiated respiratory epithelial cells are models of choice to investigate how respiratory viruses interact with the epithelial barrier when they enter the airways. They comprise cell types like ciliated cells that are important for airway function and cannot be cultured as immortalized cells. Using these culture systems, it is possible to determine which of the different cell types is susceptible to infection. These results may be different from those obtained with immortalized cells. A striking example is BRSV which infects almost all immortalized cells [18,23], whereas airway epithelial cells are rather refractory to infection ([7], this paper). Membrane-grown respiratory epithelial cells cultured under air-liquid interface conditions undergo mucociliary differentiation to generate a pseudostratified epithelium including ciliated cells, secretory cells and progenitor cells. They allow targeted access of reagents or microorganisms to the apical and basal membrane. Though we succeeded in establishing ALI cultures from bovine airway epithelial cells, most studies were performed so far with human cells. Establishing ALI cultures for cells of different species is still challenging [24,25]. The alternative culture system for differentiated respiratory epithelial cells, PCLS, can be applied to lungs from different species [7,8,26,27]. They allow analyzing infection of not only the epithelial cells but also of submucosal cells.

BRSV, BPIV3 as well as BHV-1 are important viral pathogens within the bovine respiratory disease complex [9,12]. Therefore, it was of interest to compare the entry strategies of these viruses. BPIV3 efficiently infected the airway epithelial cells using sialic acids on the apical surface as a receptor determinant for virus entry from the apical side. As virus egress also proceeds via the apical plasma membrane, BPIV3 - like human PIV3 (HPIV3) - has the characteristics of several other viruses that cause a localized infection of the respiratory tract, e.g. influenza viruses [3,28,29]. It remains to be further elucidated why BPIV3 preferentially targets the ciliated cells. This is a question that can be addressed in the future with culture systems for differentiated airway epithelial cells.

BHV-1 showed a completely different infection pattern. When BHV-1-GFP was applied to the apical side of the well-differentiated airway epithelial cells, infected cells were detected only occasionally. Recently, an explant culture system derived from the upper trachea of cows was used to analyze BHV-1 infection. Single sites of infection were detected in the epithelium when the virus was applied at 10^7^ TCID_50_/mL [30]. This infectious dose is about 100-fold higher than that required for efficient infection by BPIV3. Thus, differentiated epithelial cells from both the proximal trachea and the bronchi are rather resistant to infection by BHV-1. Successful infection required the access to the basal side of the epithelium either after opening of the tight junctions by calcium depletion or after mechanical injuring of the cell monolayer. Infection studies with ALI cultures indicated that the cells that are sensitive to BHV-1-GFP infection are not the differentiated epithelial cells but cells with the phenotype of basal cells, i.e. the progenitor cells of the differentiated epithelial cells. Also in PCLS, basal cells were found to be the primary site of infection. When the inoculation time was increased up to one week, virus was also found in the periphery of the slice, and the distribution pattern suggests direct cell-to-cell spread. This infection pattern could not be predicted from studies with polarized immortalized cells. Our results most closely resemble those reported for polarized MDCK cells where infection was not possible via the apical surface, but via the basolateral side after opening of the tight junctions or after injuring the cell monolayer [31,32]. However, this cell system could not reveal the preference for the basal cell phenotype. Other studies with polarized cells reported virus entry from both sides [33,34] or preferentially via the apical membrane [35]. These conflicting reports underline the importance of infection studies with differentiated airway epithelial cells. From our results, the question arises how BHV-1 gets across the epithelial barrier. A model that has been proposed for HSV-1 suggests entry through small lesions within the epithelium [31,32,34,36]. Further possible explanations will be discussed below.

A third infection pattern was observed when differentiated epithelial cells were infected with BRSV-GFP. Though BRSV-GFP - like BPIV3 - preferentially infected ciliated cells, the overall efficiency of infection was much lower. Whereas BPIV3 readily infected airway epithelial cells, only single BRSV-infected cells were observed even when the virus was applied at an increased MOI. The difference in infection efficiency was also evident from the amount of infectious virus released by the infected cultures. The titers of infectious progeny BRSV-GFP were up to 600-fold lower when compared to BPIV3. In contrast to BHV-1-GFP infection, the relative resistance of the airway epithelium against BRSV-GFP infection could not be overcome by opening the tight junctions or by injuring the cell monolayer. The infection pattern observed with BRSV-GFP resembles that reported for HRSV. A previous study reporting the release of a substantial amount of progeny virus had applied an unusual high multiplicity of infection [37]. Later on, it was reported also for HRSV that airway epithelial cells are infected with low efficiency [38,39]. A striking feature of the BRSV infection is the susceptibility of submucosal cells to infection. Some of these cells have morphologies that resemble those of dendritic cells. Others were located within the epithelium lining the alveolar lumen. Replication of BRSV within the alveolar epithelium in both type I and type II pneumocytes has been reported in an ultrastructural analysis of BRSV-infected calves [40]. Staining of PCLS for the presence of anti-TTF-1, which was identified as a marker for type II pneumocytes in sheep and swine [41,42], demonstrated that type II pneumocytes are susceptible to infection by BRSV-GFP. Alveolar cells that were negative for TTF-1 were also infected by BRSV-GFP. These latter cells had a flattened morphology that is characteristic for type I pneumocytes. Therefore, both type I and type II pneumocytes appear to be susceptible to infection by BRSV-GFP. Unfortunately, the identity of the infected cells in the submucosal area could not be determined. Immunological reagents available for bovine cells are limited and those few that were suitable for staining of PCLS did not provide an unambiguous result.

As differentiated airway epithelial cells are refractory to infection by BRSV-GFP and BHV-1-GFP, it remains to be elucidated how these viruses get across the epithelial barrier. As mentioned above, passage through leaky sites within the epithelial monolayer is a possibility proposed for HSV-1. An alternative explanation has been suggested for measles virus. Here, the major receptor, SLAM, is present on lymphoid cells but absent from epithelial cells. The recently identified measles receptor on epithelial cells, nectin 4, has a polarized surface distribution enabling infection via the basolateral side but not via the apical plasma membrane. Therefore, it has been suggested that measles virus infection is initiated in alveolar macrophages or dendritic cells which are used as a ferry through the epithelial barrier [43,44]. We cannot exclude that dendritic cells that uptake antigen from the airway lumen via their dendrites capture BRSV and carry infection to the submucosal infection sites as well. In-vitro studies have demonstrated RSV infection and replication within dendritic cells [45]. Also it has been suggested that the virus may persist in lymphoid or other tissues [12,46,47]. A further way to get across the epithelium without infecting the epithelial cells is transcytosis. This entry option has been shown for several viruses, e.g. for Epstein-Barr virus to get across the oral epithelium ([48], and references therein). There are no data available supporting transcytosis as an entry strategy for BHV-1 and BRSV. An alternative explanation of the infection pattern of BRSV and BHV-1 may be that the viruses enter differentiated bronchial epithelial cells without immediate initiation of replication. We do not have any evidence for this possibility, because BRSV and BHV-1 infection of differentiated bronchial epithelial cells was not enhanced even when the infection time was extended to seven days.

We would like to suggest another entry strategy that may apply to BRSV and BHV-1. Both viruses have been associated with the bovine respiratory disease complex, a multifactorial disease that involves the interplay of different factors such as stress and microbial co-infections. Bacterial pathogens associated with BRD are *Mannheimia haemolytica, Pasteurella multocida* or *Mycoplasma bovis*[12,49,50]. Infection of the epithelium by either of these microorganisms may induce a response reaction in the epithelial cells that make them susceptible to virus infection. For example, if the cells are responding by expressing suitable virus receptors that are otherwise absent from the cell surface, BRSV or BHV-1 may initiate infection. Thus, in the future it will be interesting to analyze the interaction of bacterial pathogens with differentiated airway epithelial cells and to determine whether bacterial infection predisposes the epithelium to virus infection. The analysis of the entry mechanism of the three bovine viruses is hampered by the fact that suitable antibodies against bovine nectin-1, the receptor for BHV-1, are not available commercially and the receptor for BRSV has not been identified. However, when bacterial co-infection increases the susceptibility to infection by BRSV, this approach may help to identify the receptor for BRSV. Also, our finding that pneumocytes are susceptible to BRSV-infection, may be helpful in this respect. Taken together, our data provide new insights in the airway infection by bovine respiratory viruses that may help not only to understand the pathogenicity of viruses involved in BRDC but also to develop new intervention strategies.

## Competing interests

The authors declare that they have no competing interests.

## Authors’ contributions

JK and GH conceived and designed the experiments; JK, SU and KG performed the experiments; JK, SU, GK, KG and GH analyzed the data; GK contributed reagents/materials/analysis tools; JK and GH wrote the paper. All authors read and approved the final manuscript.

## Supplementary Material

Additional file 1 **Infection of well-differentiated BAEC by BHV-1-GFP at different MOIs.** BHV-1-GFP was applied to the apical surface of ALI cultures at an MOI of 0.1, 1.0 or 10 for 2 h. Cultures were fixed at 1 – 5 dpi. Virus-infected cells are shown in green. Click here for file

Additional file 2 **TEER time course of infected ALI cultures.** Cultured cells were apically infected with either of the three viruses or mock-infected and the transepithelial resistance was measured before infection, post infection and further at daily intervals (A). TEER was also measured in EGTA-treated cultures before infection, directly after EGTA treatment, and at the indicated time points post-infection (B). Values were corrected for the blank resistance. Mean values and standard deviation of three independent cultures are shown. Click here for file

Additional file 3 **TTF-1 labeling of PCLS.** BRSV-infected PCLS were stained for TTF-1 followed by a mouse-Cy3 second antibody (red). Upper panels show a TTF-1 positive, infected cell typically located at the intersection of two or three alveoli. The middle panel represents an example of an infected cell with TTF-1 negative staining and a characteristic flattened shape. Lower panels show TTF-1 positive but uninfected control samples. Infected cells are shown in green. Nuclei were stained by DAPI. Scale bar = 50 μm. Click here for file

## References

[B1] Garibaldi RA Epidemiology of community-acquired respiratory tract infections in adults. Incidence, etiology, and impact Am J Med 1985 78 32 37 10.1016/0002-9343(85)90361-4 4014285 PMC7119376

[B2] Matrosovich MN Matrosovich TY Gray T Roberts NA Klenk HD Human and avian influenza viruses target different cell types in cultures of human airway epithelium Proc Natl Acad Sci U S A 2004 101 4620 4624 10.1073/pnas.0308001101 15070767 PMC384796

[B3] Zhang L Bukreyev A Thompson CI Watson B Peeples ME Collins PL Pickles RJ Infection of ciliated cells by human parainfluenza virus type 3 in an *in vitro* model of human airway epithelium J Virol 2005 79 1113 1124 10.1128/JVI.79.2.1113-1124.2005 15613339 PMC538579

[B4] Gray TE Guzman K Davis CW Abdullah LH Nettesheim P Mucociliary differentiation of serially passaged normal human tracheobronchial epithelial cells Am J Respir Cell Mol Biol 1996 14 104 112 10.1165/ajrcmb.14.1.8534481 8534481

[B5] Bals R Beisswenger C Blouquit S Chinet T Isolation and air-liquid interface culture of human large airway and bronchiolar epithelial cells J Cyst Fibros 2004 2 49 51 15463925 10.1016/j.jcf.2004.05.010

[B6] Vietmeier J Niedorf F Bäumer W Martin C Deegen E Ohnesorge B Kietzmann M Reactivity of equine airways – a study on precision-cut lung slices Vet Res Commun 2007 31 611 619 10.1007/s11259-007-3501-y 17252319

[B7] Goris K Uhlenbruck S Schwegmann-Wessels C Köhl W Niedorf F Stern M Hewicker-Trautwein M Bals R Taylor G Braun A Bicker G Kietzmann M Herrler G Differential sensitivity of differentiated epithelial cells to respiratory viruses reveals different viral strategies of host infection J Virol 2009 83 1962 1968 10.1128/JVI.01271-08 19052091 PMC2643795

[B8] Abd El Rahman S Winter C El-Kenawy A Neumann U Herrler G Differential sensitivity of well-differentiated avian respiratory epithelial cells to infection by different strains of infectious bronchitis virus J Virol 2010 84 8949 8952 10.1128/JVI.00463-10 20538853 PMC2919026

[B9] Snowder GD Van Vleck LD Cundiff LV Bennett GL Bovine respiratory disease in feedlot cattle: environmental, genetic and economic factors J Anim Sci 2006 84 1999 2008 10.2527/jas.2006-046 16864858

[B10] Valarcher JF Taylor G Bovine respiratory syncytial virus infection Vet Res 2007 38 153 180 10.1051/vetres:2006053 17257568

[B11] Jones C Chowdhury S A review of the biology of bovine herpesvirus type 1 (BHV-1), its role as a cofactor in the bovine respiratory disease complex and development of improved vaccines Anim Health Res Rev 2007 8 187 205 10.1017/S146625230700134X 18218160

[B12] Ellis JA Update on viral pathogenesis in BRD Anim Health Res Rev 2009 10 149 153 10.1017/S146625230999020X 20003652

[B13] Scheid A Caliguiri LA Compans RW Choppin PW Isolation of paramyxovirus glycoproteins: association of both hemagglutinating and neuraminidase activities with the larger SV5 glycoprotein Virology 1972 50 640 652 10.1016/0042-6822(72)90418-7 4118317

[B14] Chanock RM Murphy BR Collins PL Knipe DM, Howley PM Parainfluenza Viruses Fields Virology. Volume 1 2001 4 Philadelphia, PA: Lippincott/The Williams & Wilkins Co 1341 1380

[B15] Krusat T Streckert HJ Heparin-dependent attachment of respiratory syncytial virus (RSV) to host cells Arch Virol 1997 142 1247 1254 10.1007/s007050050156 9229012

[B16] Feldman SA Audet S Beeler JA The fusion glycoprotein of human respiratory syncytial virus facilitates virus attachment and infectivity via an interaction with cellular heparan sulfate J Virol 2000 74 6442 6447 10.1128/JVI.74.14.6442-6447.2000 10864656 PMC112152

[B17] Techaarpornkul S Collins PL Peeples ME Respiratory syncytial virus with the fusion protein as its only viral glycoprotein is less dependent on cellular glycosaminoglycans for attachment than complete virus Virology 2002 294 296 304 10.1006/viro.2001.1340 12009871

[B18] Tayyari F Marchant D Moraes TJ Duan W Mastrangelo P Hegele RG Identification of nucleolin as a cellular receptor for human respiratory syncytial virus Nat Med 2011 17 1132 1135 10.1038/nm.2444 21841784

[B19] Spear PG Eisenberg RJ Cohen GH Three classes of cell surface receptors for alphaherpesvirus entry Virology 2000 275 1 8 10.1006/viro.2000.0529 11017782

[B20] Spear PG Longnecker R Herpesvirus entry: an update J Virol 2003 77 10179 10185 10.1128/JVI.77.19.10179-10185.2003 12970403 PMC228481

[B21] Keil GM Fusion of the green fluorescent protein to amino acids 1 to 71 of bovine respiratory syncytial virus glycoprotein G directs the hybrid polypeptide as a class II membrane protein into the envelope of recombinant bovine herpesvirus-1 J Gen Virol 2000 81 1051 1055 10725432 10.1099/0022-1317-81-4-1051

[B22] Hajj R Baranek T Le Naour R Lesimple P Puchelle E Coraux C Basal cells of the human adult airway surface epithelium retain transit-amplifying cell properties Stem Cells 2007 25 139 148 10.1634/stemcells.2006-0288 17008423

[B23] Hoffmann M Müller MA Drexler JF Glende J Erdt M Gützkow T Losemann C Binger T Deng H Schwegmann-Weßels C Esser KH Drosten C Herrler G Differential sensitivity of bat cells to infection by enveloped RNA viruses: coronaviruses, paramyxoviruses, filoviruses, and influenza viruses PLoS One 2013 8 e72942 10.1371/journal.pone.0072942 24023659 PMC3758312

[B24] Lam E Ramke M Groos S Warnecke G Heim A A differentiated porcine bronchial epithelial cell culture model for studying human adenovirus tropism and virulence J Virol Methods 2011 78 117 123 21907242 10.1016/j.jviromet.2011.08.025

[B25] Bateman AC Karasin AI Olsen CW Differentiated swine airway epithelial cell cultures for the investigation of influenza A virus infection and replication Influenza Other Respir Viruses 2013 7 139 150 10.1111/j.1750-2659.2012.00371.x 22530566 PMC3443301

[B26] Ebsen M Mogilevski G Anhenn O Maiworm V Theegarten D Schwarze J Morgenroth K Infection of murine precision cut lung slices (PCLS) with respiratory syncytial virus (RSV) and clamydophila pneumoniae using the Krumdieck technique Pathol Res Pract 2002 198 747 753 10.1078/0344-0338-00331 12530578

[B27] Blazejewska P Koscinski I Viegas N Anhlan D Ludwig S Schughart K Pathogenicity of different PR8 influenza A virus variants in mice is determined by both viral and host factors Virology 2011 412 36 45 10.1016/j.virol.2010.12.047 21256531

[B28] Ibricevic A Pekosz A Walter MJ Newby C Battaile JT Brown EG Holtzman MJ Brody SL Influenza virus receptor specificity and cell tropism in mouse and human airway epithelial cells J Virol 2006 80 7469 7480 10.1128/JVI.02677-05 16840327 PMC1563738

[B29] Thompson CI Barclay WS Zambon MC Pickles RJ Infection of human airway epithelium by human and avian strains of influenza a virus J Virol 2006 80 8060 8068 10.1128/JVI.00384-06 16873262 PMC1563802

[B30] Steukers L Vandekerckhove AP Van den Broeck W Glorieux S Nauwynck HJ Comparative analysis of replication characteristics of BoHV-1 subtypes in bovine respiratory and genital mucosa explants: a phylogenetic enlightenment Vet Res 2011 42 33 10.1186/1297-9716-42-33 21324115 PMC3050707

[B31] Schelhaas M Jansen M Haase I Knebel-Mörsdorf D Herpes simplex virus type 1 exhibits a tropism for basal entry in polarized epithelial cells J Gen Virol 2003 84 2473 2484 10.1099/vir.0.19226-0 12917468

[B32] Marozin S Prank U Sodeik B Herpes simplex virus type 1 infection of polarized epithelial cells requires microtubules and access to receptors present at cell-cell contact sites J Gen Virol 2004 85 775 786 10.1099/vir.0.19530-0 15039520

[B33] Sears AE McGwire BS Roizman B Infection of polarized MDCK cells with herpes simplex virus 1: two asymmetrically distributed cell receptors interact with different viral proteins Proc Natl Acad Sci USA 1991 88 5087 5091 10.1073/pnas.88.12.5087 1647025 PMC51816

[B34] Tran LC Kissner JM Westerman LE Sears AE A herpes simplex virus 1 recombinant lacking the glycoprotein G coding sequences is defective in entry through apical surfaces of polarized epithelial cells in culture and *in vivo* Proc Natl Acad Sci USA 2000 97 1818 1822 10.1073/pnas.020510297 10677539 PMC26519

[B35] Galen B Cheshenko N Tuyama A Ramratnam B Herold BC Access to nectin favors herpes simplex virus infection at the apical surface of polarized human epithelial cells J Virol 2006 80 12209 12218 10.1128/JVI.01503-06 17005657 PMC1676285

[B36] Yoon M Spear PG Disruption of adherens junctions liberates nectin-1 to serve as receptor for herpes simplex virus and pseudorabies virus entry J Virol 2002 76 7203 7208 10.1128/JVI.76.14.7203-7208.2002 12072519 PMC136315

[B37] Zhang L Peeples ME Boucher RC Collins PL Pickles RJ Respiratory syncytial virus infection of human airway epithelial cells is polarized, specific to ciliated cells, and without obvious cytopathology J Virol 2002 76 5654 5666 10.1128/JVI.76.11.5654-5666.2002 11991994 PMC137037

[B38] Wright PF Ikizler MR Gonzales RA Carroll KN Johnson JE Werkhaven JA Growth of respiratory syncytial virus in primary epithelial cells from the human respiratory tract J Virol 2005 79 8651 8654 10.1128/JVI.79.13.8651-8654.2005 15956607 PMC1143745

[B39] Villenave R Thavagnanam S Sarlang S Parker J Douglas I Skibinski G Heaney LG McKaigue JP Coyle PV Shields MD Power UF *In vitro* modeling of respiratory syncytial virus infection of pediatric bronchial epithelium, the primary target of infection *in vivo* Proc Natl Acad Sci USA 2012 109 5040 5045 10.1073/pnas.1110203109 22411804 PMC3323997

[B40] Bryson DG McConnell S McAliskey M McNulty MS Ultrastructural features of alveolar lesions in induced respiratory syncytial virus pneumonia of calves Vet Pathol 1991 28 286 292 10.1177/030098589102800404 1949507

[B41] Beytut E Sheep pox virus induces proliferation of type II pneumocytes in the lungs J Comp Pathol 2010 143 132 141 10.1016/j.jcpa.2010.01.014 20181359

[B42] Balka G Ladinig A Ritzmann M Saalmüller A Gerner W Käser T Jakab C Rusvai M Weißenböck H Immunohistochemical characterization of type II pneumocyte proliferation after challenge with type I porcine reproductive and respiratory syndrome virus J Comp Pathol 2013 149 322 330 10.1016/j.jcpa.2012.12.006 23453491

[B43] Lemon K de Vries RD Mesman AW McQuaid S van Amerongen G Yüksel S Ludlow M Rennick LJ Kuiken T Rima BK Geijtenbeek TB Osterhaus AD Duprex WP de Swart RL Early target cells of measles virus after aerosol infection of non-human primates PLoS Pathog 2011 7 e1001263 10.1371/journal.ppat.1001263 21304593 PMC3029373

[B44] Antunes Ferreira CS Frenzke M Leonard VHJ Welstead GG Richardson CD Cattaneo R Measles virus infection of alveolar macrophages and dendritic cells precedes spread to lymphatic organs in transgenic mice expressing human signaling lymphocytic activation molecule (SLAM, CD150) J Virol 2010 84 3033 3042 10.1128/JVI.01559-09 20042501 PMC2826031

[B45] de Graaff PM de Jong EC van Capel TM van Dijk ME Roholl PJ Boes J Luytjes W Kimpen JL van Bleek GM Respiratory syncytial virus infection of monocyte-derived dendritic cells decreases their capacity to activate CD4 T cells J Immunol 2005 175 5904 5911 16237083 10.4049/jimmunol.175.9.5904

[B46] De Jong MC van der Poel WH Kramps JA Brand A van Oirschot JT Quantitative investigation of population persistence and recurrent outbreaks of bovine respiratory syncytial virus on dairy farms Am J Vet Res 1996 57 628 633 8723872

[B47] Valarcher JF Bourhy H Lavenu A Bourges-Abella N Roth M Andreoletti O Ave P Schelcher F Persistent infection of B lymphocytes by bovine respiratory syncytial virus Virology 2001 291 55 67 10.1006/viro.2001.1083 11878876

[B48] Tugizov SM Herrera R Palefsky JM Epstein-Barr virus transcytosis through polarized oral epithelial cells J Virol 2013 87 8179 8194 10.1128/JVI.00443-13 23698302 PMC3700193

[B49] Srikumaran S Kelling CL Ambagala A Immune evasion by pathogens of bovine respiratory disease complex Anim Health Res Rev 2007 8 215 229 10.1017/S1466252307001326 18218162

[B50] Rice JA Carrasco-Medina L Hodgins DC Shewen PE Mannheimia haemolytica and bovine respiratory disease Anim Health Res Rev 2007 8 117 128 10.1017/S1466252307001375 18218156

