# Supplementary figures and images for: Three viruses of the bovine respiratory disease complex apply different strategies to initiate infection
Source: Vet Res. 2014 Feb 18;45(1):20. doi: 10.1186/1297-9716-45-20 (PMC3942114; doi:10.1186/1297-9716-45-20)

day 1

day 2

day 3

day 4

day 5

MOI ~ 0.1

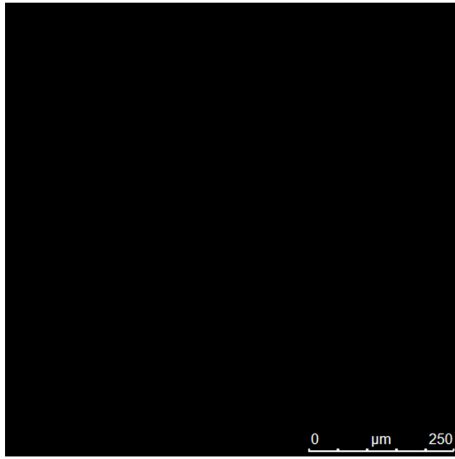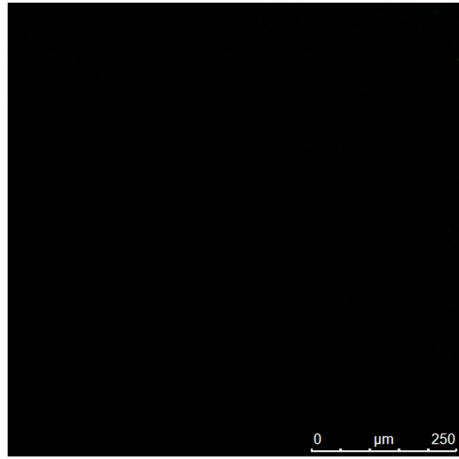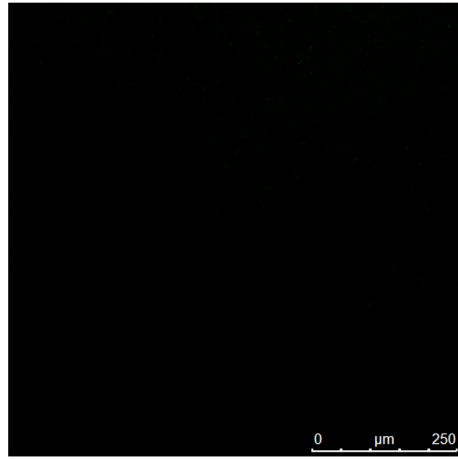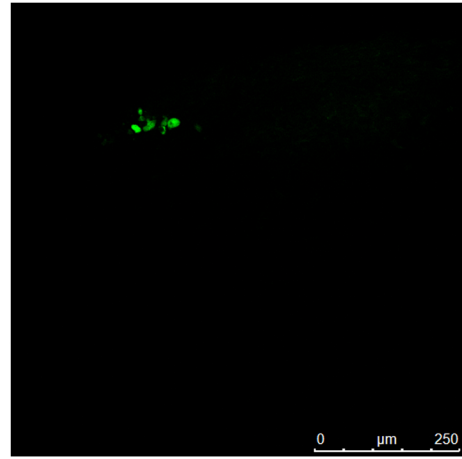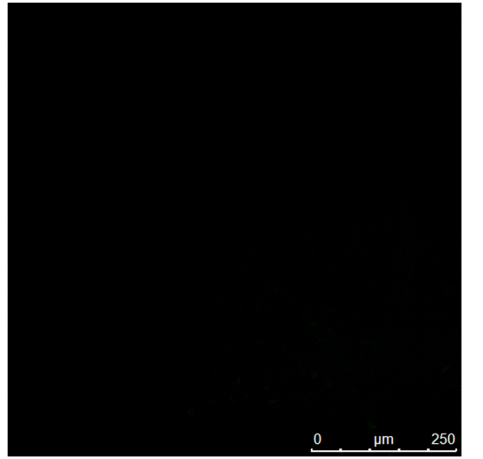

MOI ~ 1.0

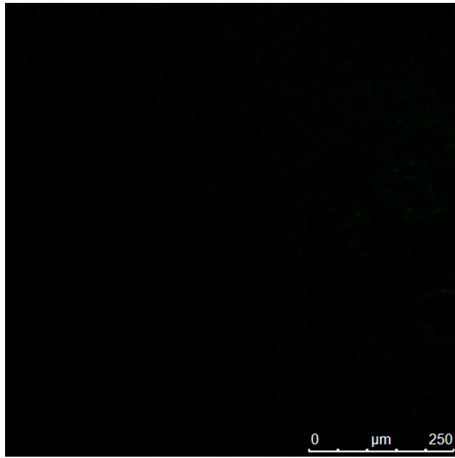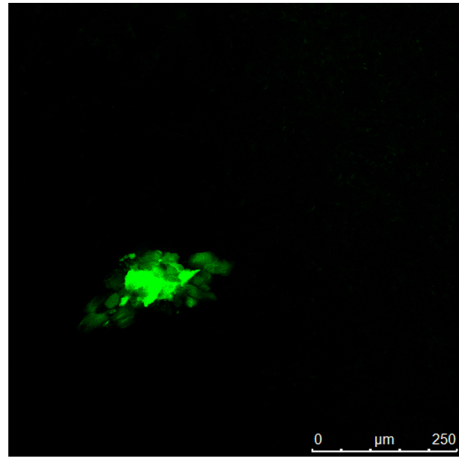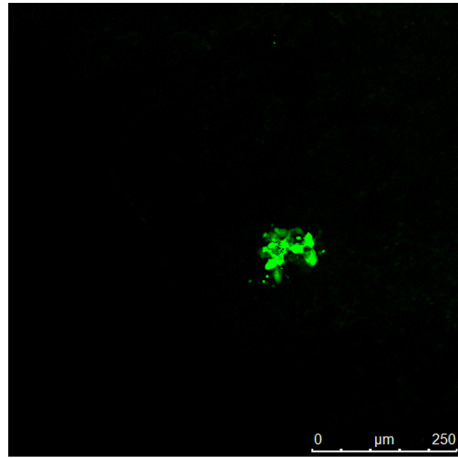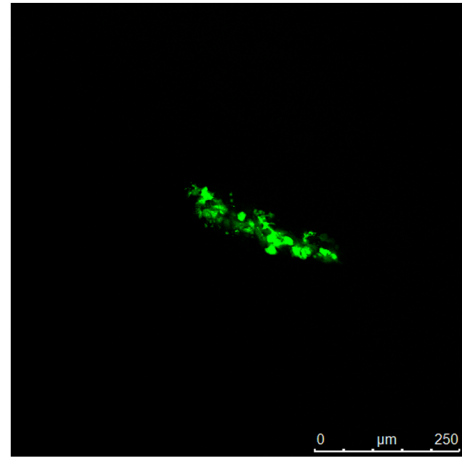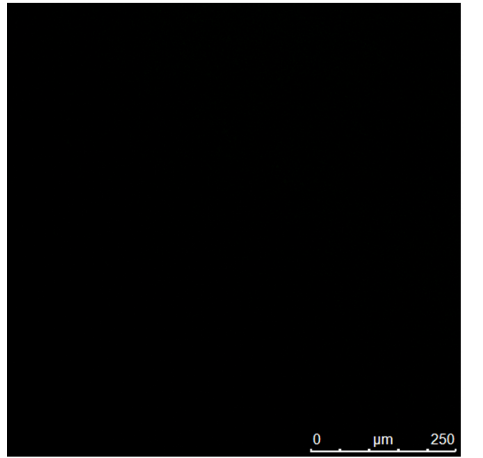

MOI ~ 10

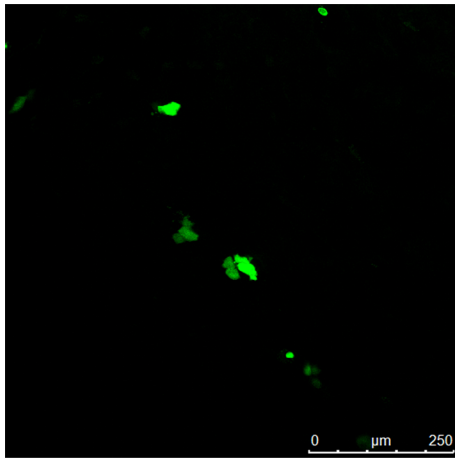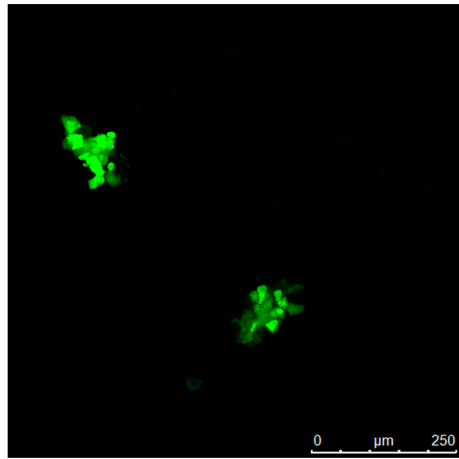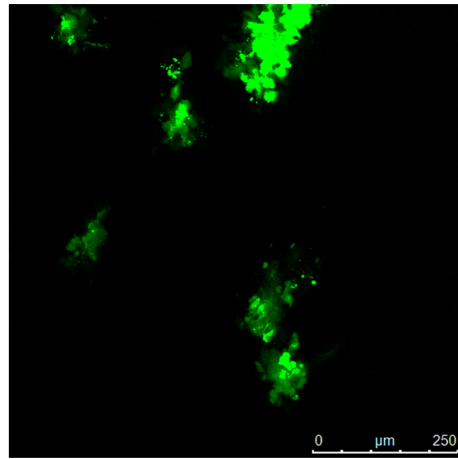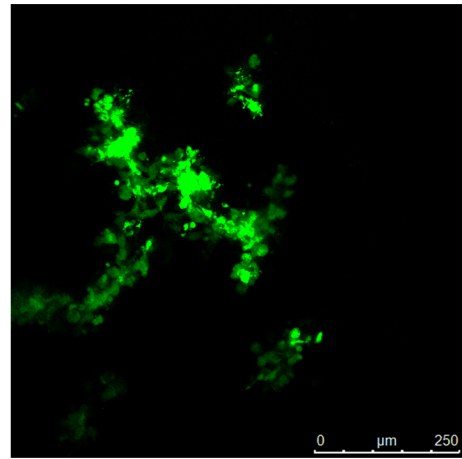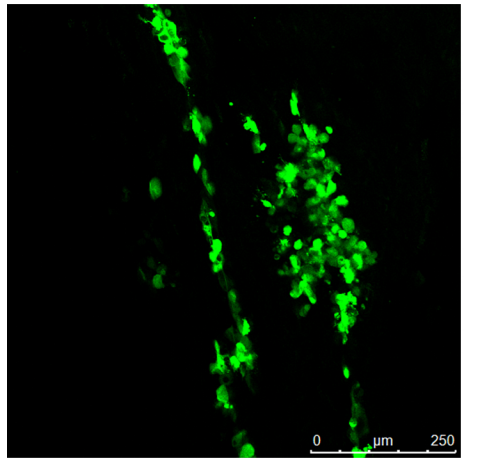

Supplement: Additional file 1 — Infection of well-differentiated BAEC by BHV-1-GFP at different MOIs. BHV-1-GFP was applied to the apical surface of ALI cultures at an MOI of 0.1, 1.0 or 10 for 2 h. Cultures were fixed at 1 – 5 dpi. Virus-infected cells are shown in green. [file 1297-9716-45-20-S1.pdf]

**A**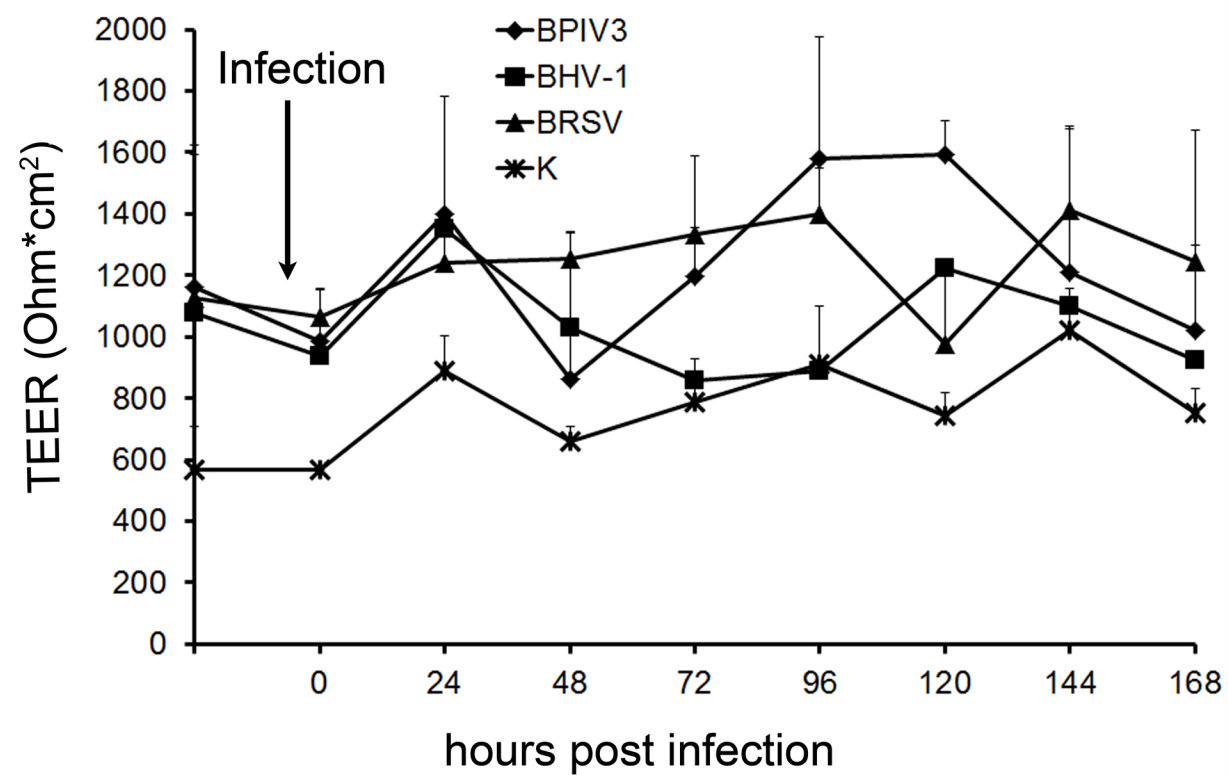**B**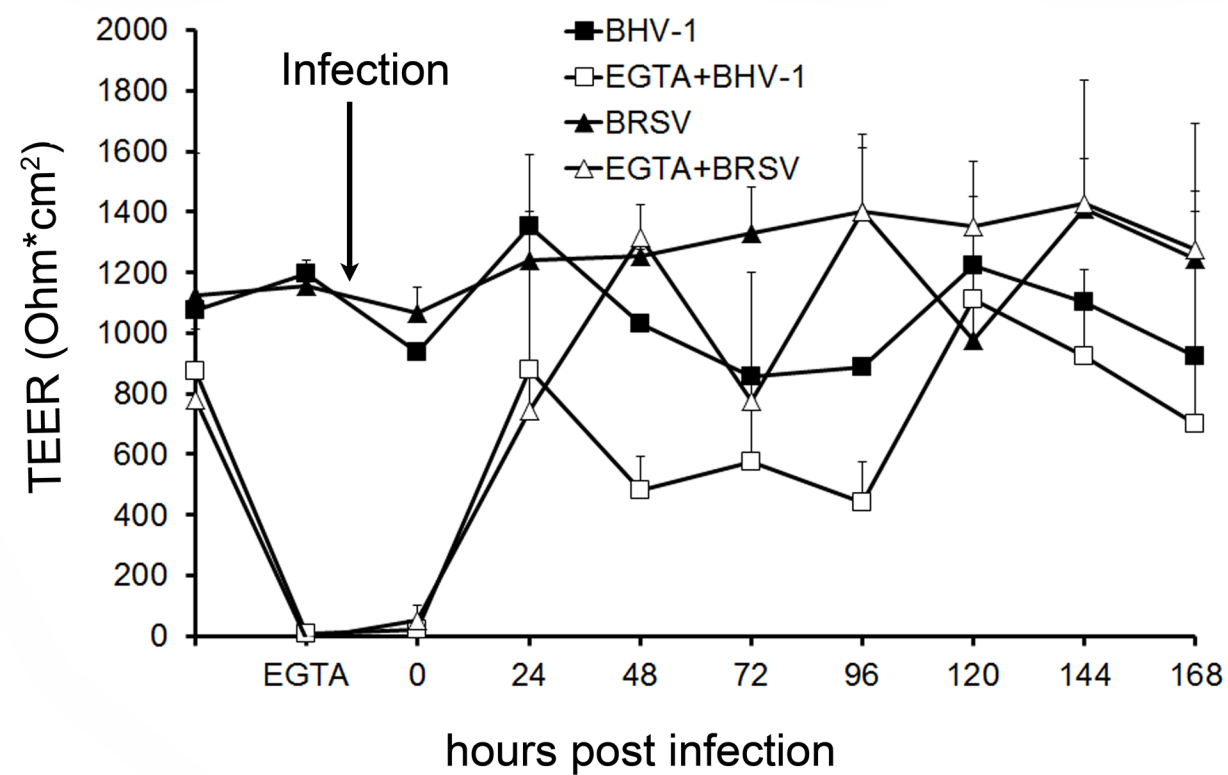

Supplement: Additional file 2 — TEER time course of infected ALI cultures. Cultured cells were apically infected with either of the three viruses or mock-infected and the transepithelial resistance was measured before infection, post infection and further at daily intervals (A). TEER was also measured in EGTA-treated cultures before infection, directly after EGTA treatment, and at the indicated time points post-infection (B). Values were corrected for the blank resistance. Mean values and standard deviation of three independent cultures are shown. [file 1297-9716-45-20-S2.pdf]

TTF-1

BRSV-GFP

DAPI

overlay

TTF-1-positive

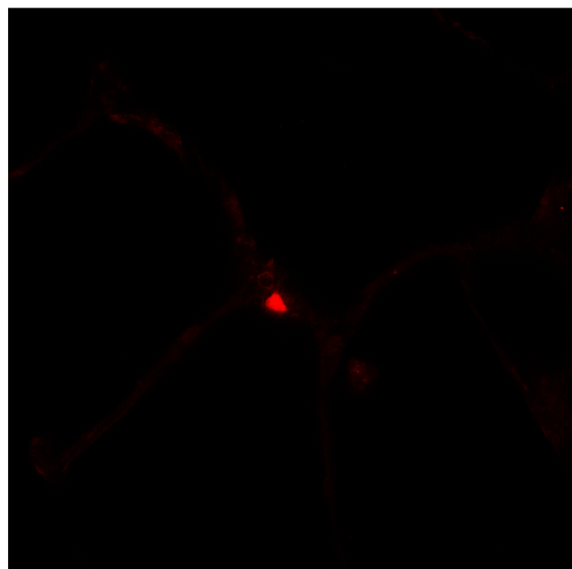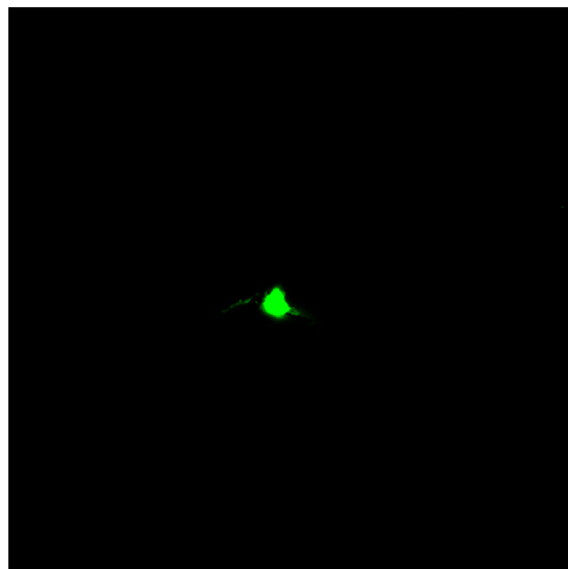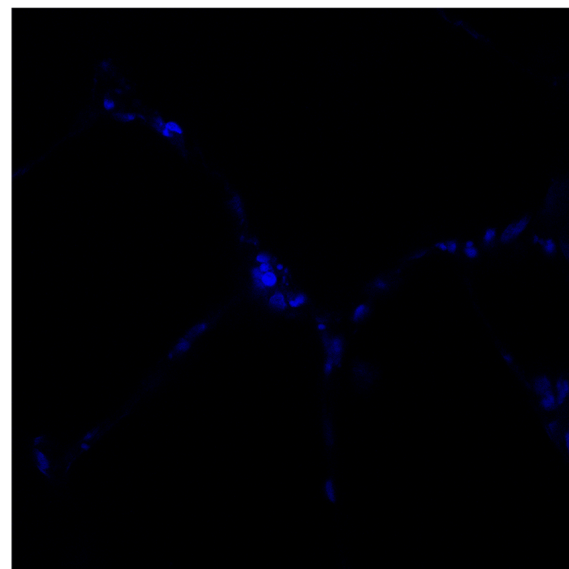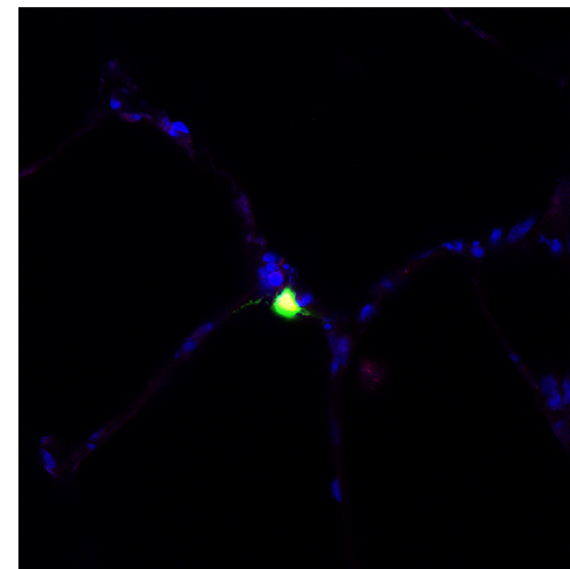

TTF-1-negative

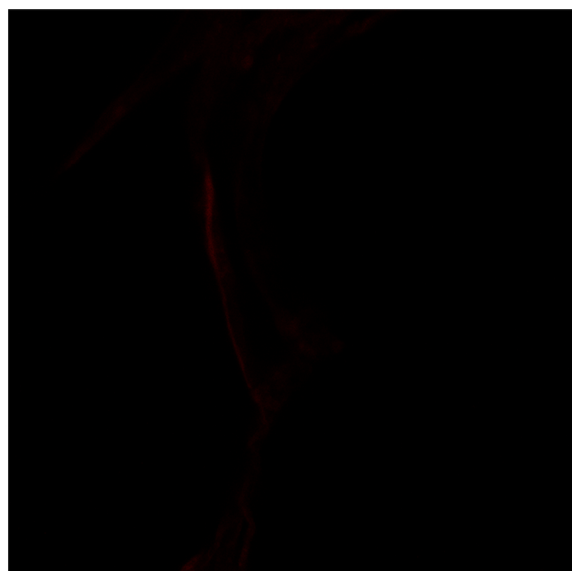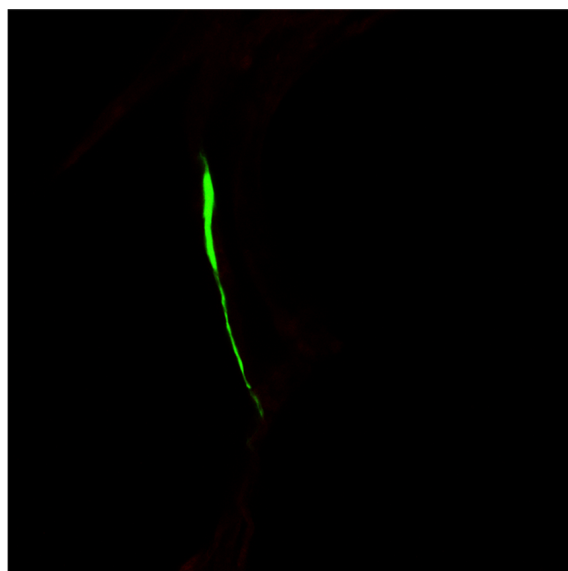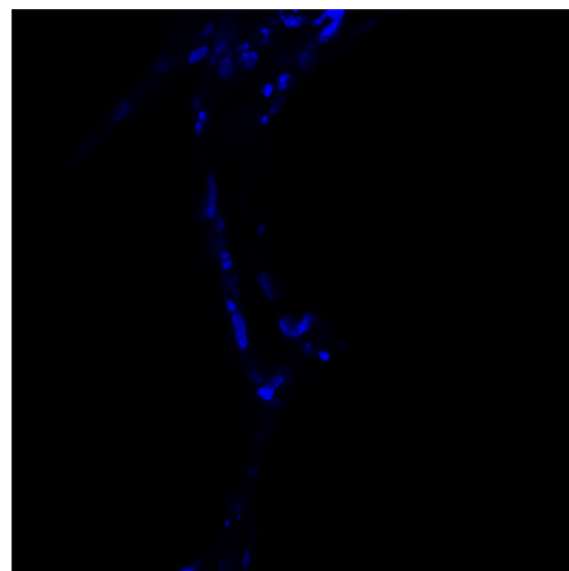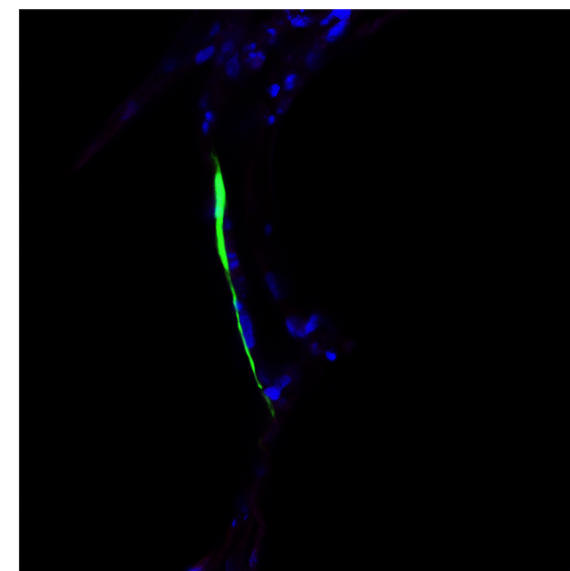

control

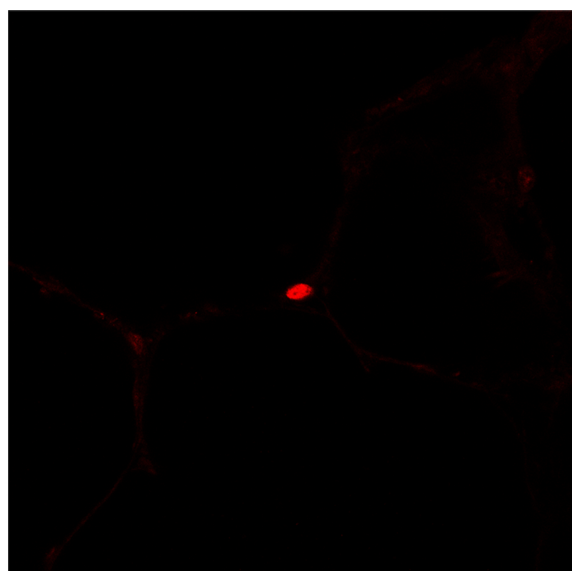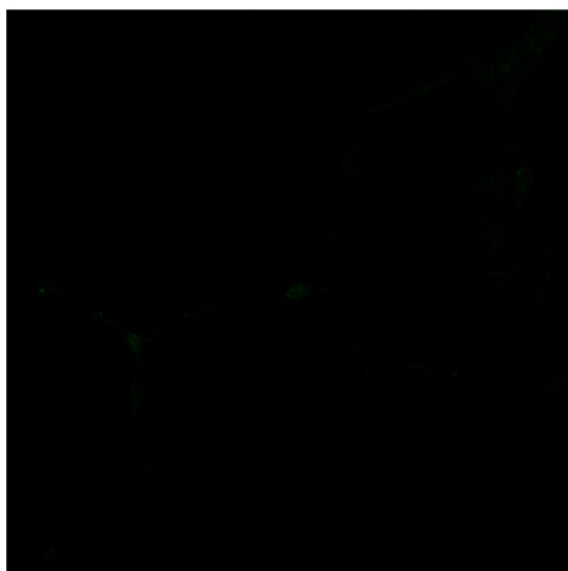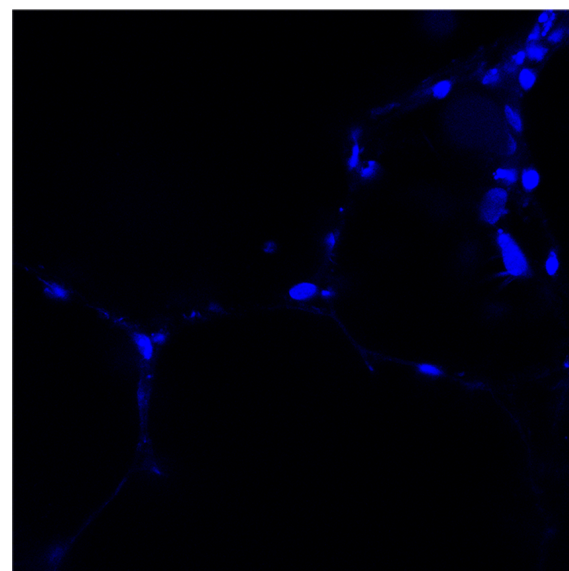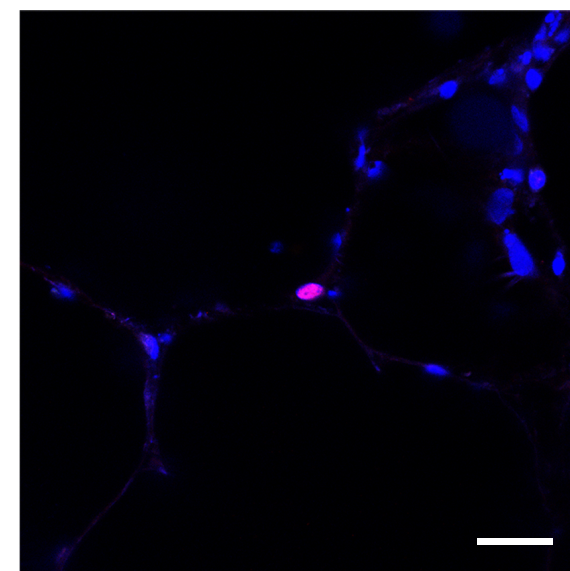

Supplement: Additional file 3 — TTF-1 labeling of PCLS. BRSV-infected PCLS were stained for TTF-1 followed by a mouse-Cy3 second antibody (red). Upper panels show a TTF-1 positive, infected cell typically located at the intersection of two or three alveoli. The middle panel represents an example of an infected cell with TTF-1 negative staining and a characteristic flattened shape. Lower panels show TTF-1 positive but uninfected control samples. Infected cells are shown in green. Nuclei were stained by DAPI. Scale bar = 50 μm. [file 1297-9716-45-20-S3.pdf]
